# Ernährungspraxis auf Intensivstationen: nutritionDay 2007–2021

**DOI:** 10.1007/s00063-023-00996-y

**Published:** 2023-02-28

**Authors:** Michael Hiesmayr, Arabella Fischer, Cecilia Veraar, Bruno Mora, Silvia Tarantino, Arved Weimann, Dorothee Volkert

**Affiliations:** 1 Zentrum für Medical Data Science, Medizinische Universität Wien, Wien, Österreich; 2 Klinische Abteilung Herz-Thorax-Gefäßchirurgische Anästhesie und Intensivmedizin, Medizinische Universität Wien, Spitalgasse 23, 1090 Wien, Österreich; 3 Abteilung für Allgemein‑, Viszeral- und Onkologische Chirurgie, Klinikum St. Georg, Leipzig, Deutschland; 4 Institut für Biomedizin des Alterns, Friedrich-Alexander-Universität, Erlangen-Nürnberg, Nürnberg, Deutschland

**Keywords:** Ernährungstherapie, Intensivmedizin, Parenterale Ernährung, Enterale Ernährung, Aufenthaltsdauer, Nutrition therapy, Intensive care, Parenteral nutrition, Enteral nutrition, Length of stay

## Abstract

Die klinische Praxis der Ernährungstherapie auf Intensivstationen, eine anerkannte Unterstützung von Patient*innen, die nicht selbst ausreichend essen können, basiert auf teils divergierenden Leitlinien und randomisierten Studien, die unterschiedliche Vorgangsweisen zum Standard erhoben. Zweck der aktuellen Analyse von Daten des nutritionDay-Projekts ist es, die derzeit weltweite klinische Praxis mit jener in Europa sowie in der Region Deutschland, Österreich und Schweiz (DACH) zu vergleichen. Zwischen den Jahren 2007 und 2021 wurden Daten von 18.918 Patient*innen, die in 1595 Intensivstationen aufgenommen waren, in einer Querschnittsuntersuchung online erfasst. Die Untersuchung verfolgt das Ziel, alle an einem Tag anwesenden Patient*innen zu erfassen. Die mediane Aufenthaltsdauer bei der Erfassung war Tag 4. Patient*innen unterschieden sich gering zwischen den Regionen mit einem medianen Alter von 64 Jahren und einem Frauenanteil von 40 %. Die Patient*innen waren zur Hälfte beatmet, in 29 % sediert und erhielten in 10 % eine Nierenersatztherapie. Nach 60 Tagen war die Hälfte nach Hause entlassen und ein Viertel der Patient*innen verstorben. Die Ernährungstherapie wird doppelt so häufig in Form enteraler Ernährung (48 %) im Vergleich zur parenteralen Ernährung (24 %) verabreicht. Etwa 39 % der Patient*innen können essen und 10 % erhalten keine Ernährung. Parenterale Ernährung wird in Europa deutlich häufiger als in den anderen Weltregionen angewandt. Die Menge der Ernährung ist in allen Regionen sehr ähnlich mit etwa 1500 kcal und 60 g Protein pro Tag. Eine klare Beziehung mit dem Körpergewicht ist nicht erkennbar. Die Streuung um diese medianen Werte ist sehr groß mit jeweils mehr als der Hälfte der Patient*innen, die um mehr als 25 % nach oben oder unten abweichen. Das nutritionDay-Projekt erlaubt jeder Intensivstation, ihre Praxis numerisch und grafisch mit den durchschnittlichen Werten der weltweiten Daten im Sinne des Benchmarkings jährlich zu vergleichen. Ziel ist es, die Heterogenität der Praxis zu vermindern.

## Abstract (en)

In intensive care units (ICU), patients who are not able to eat or are considered at nutritional risk typically receive medical nutrition therapy based on partially contradictory guidelines as well as the strategies used in large randomized trials. The aim of this study is to analyze patient data from the nutritionDay project in intensive care to describe current clinical approaches to nutrition support worldwide, in Europe and in the group of German-speaking countries, the DACH (i.e., Germany, Austria, Switzerland) region. From 2007–2021, data of 18,918 adult patients in 1595 ICUs from 63 different countries were included in this cross-sectional study. The aim was to recruit all patients present in ICUs. Median stay in the ICU was 4 days on nutritionDay. Little difference in patient characteristics were observed between worldwide, Europe, and the DACH region. Patient were 64 years old, 40% female, 50% ventilated, 29% sedated, and 10% needed renal replacement therapy. A quarter of the patients died in hospital within 60 days and about half of the patients had been discharged home. Enteral nutrition was given twice as frequently as parenteral nutrition (48% versus 24%). Many patients received oral nutrition (39%) and a substantial number received no nutrition support (10%). Parenteral nutrition was used more frequently in Europe than in other world regions, the lowest use being observed in North America. The amount of nutrition given is very similar in all regions regardless of the nutrition route with about 1500 kcal and 60 g of protein per day. A clear association with body weight was not observed and the variation around the median was very large with half of patients receiving amounts 25% below or above the median. Upon completion of data entry, the nutritionDay project allows each ICU to download a unit report summarizing data that allows comparison with worldwide data in numeric and graphical form to permit easy benchmarking of medical nutrition therapy.

## Hintergrund

Grundsätzlich besteht unter Intensivmediziner*innen die Übereinstimmung, dass Patient*innen, die nicht selbst genügend essen können, eine Ernährungstherapie in Form von enteraler (EN) oder parenteraler Ernährung (PN) erhalten sollen [1–9]. Das generelle Ziel ist es, durch Ernährungstherapie dem aufgrund der kritische Erkrankung gesteigerten Katabolismus entgegenzuwirken.

Uneinigkeit herrscht über nahezu alle Details der Ernährungstherapie wie den Zeitpunkt des Beginns, die genaue Menge und Zusammensetzung im Verlauf der kritischen Erkrankung sowie den zu wählenden Zufuhrweg. Diese Uneinigkeit widerspiegelt sich in den großen randomisierten Studien [10–19] ebenso wie in den Leitlinien, die auch durch unterschiedliche Schulen beeinflusst werden.

Die nordamerikanische Schule setzt primär auf EN mit einem zunehmend höheren Proteinanteil und früherer Ernährung bei höherem Ernährungsrisiko, das an der zunehmenden Schwere der Erkrankung festgemacht wird [1, 4, 6]. Eine PN wird bei erfolgloser EN nach einer Woche und bei Mangelernährten früher empfohlen. Bei einem Body-Mass-Index (BMI) > 30 kg/m^2^ wird die Nährstoffzufuhr bei weiterhin hoher Proteinzufuhr nahezu halbiert.

Die europäische Schule setzt auf frühe Ernährung für alle, die nicht selbst essen

Die europäische Schule setzt auf frühe Ernährung für alle, die nicht selbst essen, ohne einen besonderen Bezug zum Konzept des Ernährungsrisikos [5, 7]. Die Menge der zugeführten Nährstoffe sollte dem geschätzten (20–25 kcal/kg Körpergewicht [KG]) oder besser gemessenen Nährstoffverbrauch etwa ab dem 3–5 Tag entsprechen. Über die erste Woche des Intensivaufenthalts wird die Zufuhr von etwa 70 % des geschätzten/gemessenen Nährstoffverbrauchs angestrebt. Die Proteinmenge wird mit etwa 1,3 g/kgKG empfohlen. In der europäischen Leitlinie der European Society for Clinical Nutrition and Metabolism (ESPEN) wird ab einem BMI > 25 kg/m^2^ die zugeführte Menge vom Idealgewicht abgeleitet. Das geschätzte überschüssige Fettgewebe wird lediglich mit 30 % berücksichtigt. Keine der Leitlinien nimmt einen klaren Bezug zum veränderten Bedarf an Nährstoffstoffen in Abhängigkeit von Geschlecht und Alter.

Die deutsche Leitlinie sieht 2 entscheidende Abweichungen vor [21]. Dabei wird die Menge der zugeführten Ernährung bei hohem Insulinbedarf zur Kontrolle der Hyperglykämie und bei Auftreten einer Hypophosphatämie entsprechend einem Algorithmus reduziert.

Ziel der aktuellen Analyse ist es, die weltweite Praxis der Ernährungstherapie und etwaige Abweichungen in den deutschsprachigen Ländern (Deutschland, Österreich, Schweiz: DACH) zu beschreiben. Dabei soll besonders die Personalisierung der Ernährungstherapie bezüglich Menge und Zusammensetzung analysiert werden.

## Methodik

An der Querschnittsuntersuchung nutritionDay können seit dem Jahr 2007 jährlich auch Intensivstationen teilnehmen (www.nutritionday.org). Die Datenerhebung wird mittels Fragebögen, die in 27 Sprachen verfügbar sind, durchgeführt [22]). Sie findet jeweils im November statt. Alle Patient*innen sollen in der ersten Woche inkludiert werden und können in den folgenden Wochen jeweils durch die innerhalb der letzten 6 Tage neuaufgenommenen Patient*innen ergänzt werden, um eine repräsentative Stichprobe für die individuelle Intensivstation zu erreichen. Erfasst werden die Strukturen der Intensivstation, patient*innenbezogene Daten vom Aufnahmetag und aktuellen Tag, detaillierte Informationen zur aktuellen Ernährungstherapie und das Outcome im Krankenhaus nach 60 Tagen. Der Schweregrad wird mittels Simplified-Acute-Physiology-Score 2 (SAPS2) [23], der aktuelle Schweregrad mittels Sepsis-related-organ-failure-assessment(SOFA)-Score [24] und der pflegerische Aufwand mittels Nine-equivalents-of-nursing-manpower-use-Score (NEMS; [25]) aus den Rohdaten berechnet. Die erhobenen Daten werden nach Registrierung in eine gesicherte elektronische Datenbank, die an der Medizinischen Universität Wien am Center for Medical Data Science gehostet wird, eingetragen. Die teilnehmenden Stationen können nach Abschluss der Dateneingabe einen Benchmarkingbericht downloaden, der einen Vergleich mit den weltweiten Daten der vergangenen 3 Jahre zeigt [26].

Die Daten der Querschnittserhebung sind primär als Prävalenzen zu verstehen

Die statistische Auswertung inkludiert alle erwachsenen Patient*innen älter als 17 Jahre. Die deskriptive Statistik wird für die weltweite, die europäische und die deutschsprachige Kohorte als Median mit Interquartilsabstand (IQR) oder Prozentsätzen präsentiert. Zur Beschreibung der Ernährungspraxis einzelner Stationen wird zuerst auf Stationsniveau aggregiert. Eine Sensitivitätsanalyse wird für alle Stationen, die mindestens 6 Patient*innen inkludiert und für > 80 % der Patient*innen das Outcome 60 Tage nach nutritionDay erhoben haben, durchgeführt. Vergleiche zwischen Kategorien werden mittels generalisierten linearen Modells (GLM) ermittelt, wobei individuelle Intensivstationen als Random-Faktoren betrachtet werden und Schätzungen als Odds-Ratio (OR) mit 95 %-Konfidenzintervall (95 %-KI) angegeben. Vergleiche zwischen Regionen wurden für ungeplante vs. geplante und chirurgisch vs. medizinische Aufnahme adjustiert. Alle Analysen wurden mittels Stata 15.1 (StataCorp LLC**, **College Station, TX, USA) durchgeführt. Die Daten der Querschnittserhebung sind primär als Prävalenzen zu verstehen. Die zeitliche Entwicklung der unterschiedlichen Outcomes in Abhängigkeit von der Ernährungsform zeigen kumulative Inzidenzen [27], die eine Abschätzung der tatsächlichen Outcomes erlauben.

## Ergebnisse

### Demografie und Outcome

Die nutritionDay-Kohorte aus den Jahren 2007–2021 umfasst 18.918 Patient*innen, die mindestens 18 Jahre alt sind und auf 1595 Intensivstationen in 63 Ländern, 995 aus Europa und 162 aus der DACH-Region, aufgenommen wurden. In der DACH-Region sind die Patient*innen älter (3,2 Jahre; 95 %-KI 2,1–4,3), interessanterweise seltener Frauen (OR 0,87; 95 %-KI 0,77–0,97) und zeigen häufiger chirurgische Indikationen und Herz-Kreislauf-Erkrankungen als Grund für den Intensivaufenthalt (Tab. 1). Der Schweregrad bei Aufnahme ist in Europa und in der DACH-Region gleich hoch, aber deutlich höher als in Nordamerika und Asien.

**Table 1:** 

	Weltweit		Europa		DACH	
N	18.918		11.179		1773	
	Median	IQR	Median	IQR	Median	IQR
Alter	64	52–74	65	53–75	68	56–76
Geschlecht (weiblich)	40,4 %	–	39,1 %	–	36,3 %	–
Körpergewicht (kg)	73	62–85	75	65–87	78	67–90
BMI (kg/m^2^)	25,7	22,8–29,4	26,0	23,1–29,4	26,2	23,4–29,9
Aufnahme ungeplant	64,2 %	–	64,1 %	–	65,8 %	–
Chirurgisch	43,3 %	–	47,6 %	–	65,2 %	–
*Aufnahmegrund*						
Abdomen	16,6 %	–	19,5 %	–	19,1 %	–
Respiration	28,1 %	–	28,3 %	–	24,7 %	–
Herz/Kreislauf	22,8 %	–	23,5 %	–	38,5 %	–
Zentrales Nervensystem	17,4 %	–	16,7 %	–	11,7 %	–
Sepsis	15,3 %	–	16,3 %	–	12,1 %	–
Trauma	8,7 %	–	8,6 %	–	6,6 %	–
Andere?	15,4 %	–	13,8 %	–	13,3 %	–
Beatmung	49,3 %	–	50,6 %	–	49,5 %	–
Sediert	29,0 %	–	29,4 %	–	32,9 %	–
Nierenersatztherapie	9,9 %	–	9,5 %	–	12,6 %	–
NEMS	23	15–32	24	15–24	27	15–34
SOFA-Score	4	1–6	4	2–6	4	2–7
SAPS2	38	27–52	40	29–54	41	30–55
„Predicted mortality“^a^	31 %	–	33 %	–	35 %	–
Mortalität (Tag 60)	24,6 %	–	26,0 %	–	23,8 %	–
ICU-Aufenthalt bis nutritionDay (Tage)	4	1–13	5	2–14	4	1–13
ICU-Aufenthalt (Tage)	11	4–27	12	5–29	12	4–30
Gesamter KH-Aufenthalt (Tage)	22	11–45	26	13–51	27	14–50

^a^„Predicted mortality“ ist ein Mittelwert, um einen Vergleich mit der tatsächlichen Mortalität zu ermöglichen

*BMI* Body-Mass-Index; *ICU* Intensivstation; *DACH* Deutschland, Österreich, Schweiz; *IQR* Interquartilsabstand; *KH* Krankenhaus; *NEMS* Nine-equivalents-of-nursing-manpower-use-Score; *SAPS2* Simplified-Acute-Physiology-Score 2; *SOFA* „sepsis related organ failure assessment“

Eine Beatmung wird unabhängig von den Regionen häufiger bei ungeplanten (OR 2,19; 95 %-KI 1,96–2,44) und chirurgischen Aufnahmen (OR 1,30; 95 %-KI 1,14–1,49) vorgenommen. Eine Sedierung wird ebenfalls häufiger bei ungeplanten (OR 1,91; 95 %-KI 1,70–2,15) und chirurgischen Aufnahmen (OR 1,57; 95 %-KI 1,39–1,78) vorgenommen, wobei in der DACH-Region deutlich häufiger Patient*innen sediert werden (OR 1,36; 95 %-KI 1,14–1,63). Eine Nierenersatztherapie wird häufiger bei ungeplanten Aufnahmen (OR 1,35; 95 %-KI 1,14–1,61) und seltener bei chirurgischen Aufnahmen (OR 0,79; 95 %-KI 0,67–0,94), wobei wiederum deutlich häufiger in der DACH-Region (OR 1,58; 95 %-KI 1,27–1,98) als in allen anderen Regionen notwendig. Anhand des NEMS-Scores findet sich gegenüber Europa mit 25,6 Punkten (95 %-KI 25,1–26,2) ein höherer Therapieaufwand in der DACH-Region mit 28,9 Punkten (95 %-KI 27,7–30,0) und in Nordamerika mit 28,0 Punkten (95 %-K 26,7–29,4; *p* < 0,001) nach Korrektur für ungeplante und chirurgische Aufnahmen.

In der DACH-Region werden deutlich häufiger Patient*innen sediert

Das Outcome wurde für Intensivstationen, die die Sensitivitätskriterien erfüllen, ausgewertet. Nach Hause entlassen ist nach 60 Tagen weniger als die Hälfte der Patient*innen 5836 (46 %), 2292 (18 %) sind in andere Krankenhäusern verlegt und 1205 (10 %) befinden sich noch im selben Krankenhaus, während 3065 (24 %) verstorben sind. Die Mortalität ist vom Schweregrad bei Aufnahme abhängig (SAPS2: < 24, 6 %; SAPS2: 24–33, 14 %; SAPS2: 34–42, 24 %; SAPS2: 43–54, 31 %; Saps2: 55–111: 43 %), niedriger bei chirurgischen Aufnahmen (*p* < 0,001) und in der DACH-Region (*p* < 0,05), Nordamerika (*p* < 0,05) sowie in Asien (*p* < 0,01) verglichen mit dem restlichen Europa. Die Aufenthaltsdauer auf der Intensivstation ist zwischen den Regionen vergleichbar. Die gesamte Krankenhausaufenthaltsdauer ist in Europa etwas länger als weltweit, wobei der unterschiedliche Anteil von Verlegungen in andere Krankenhäuser einen großen Einfluss haben könnte.

### Ernährungstherapie

**Figure 1:**
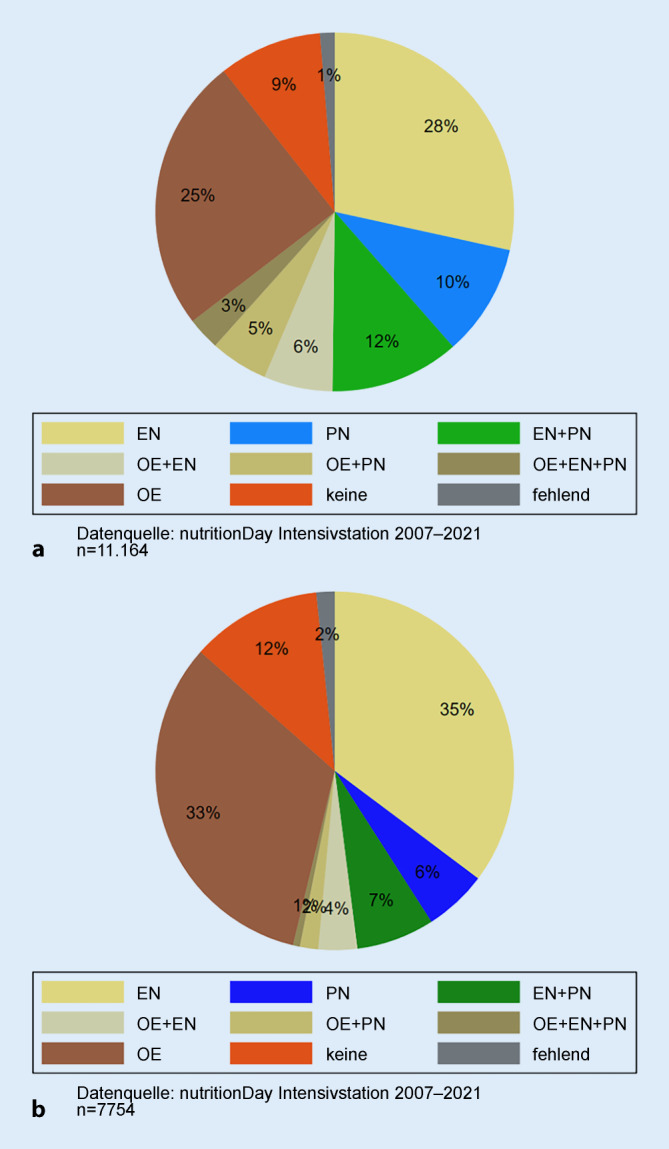

Am nutritionDay war die häufigste Ernährungsform die EN bei 9099 (49 %) der Patient*innen, gefolgt von PN bei 4505 (24 %) Patient*innen, oraler Ernährung (OE) bei 7363 (39 %) Patient*innen und keiner Ernährung bei 1956 (10 %) Patient*innen. Lediglich bei 275 (1,5 %) Patient*innen gab es keine Information zur Ernährungsform. Eine Ernährungstherapie wird auch in verschiedensten Kombinationen verabreicht, wobei generell die PN häufiger in Europa (Abb. 1a) und die EN häufiger außerhalb Europas (Abb. 1b) verabreicht wird. Auch die OE wird außerhalb Europas häufiger verwendet.

Die unterschiedliche Praxis in der Auswahl unterschiedlicher Ernährungsformen wird noch klarer, wenn man den Prozentsatz der Patient*innen auf Stationsniveau zusammenfasst. In Europa versorgen 9 % der Intensivstationen keinen Patient*in und in 7 % alle Patient*innen mit EN. Eine ähnlich große Streuung wird außerhalb Europas mit 8 % und 7 % beobachtet. Bei der PN zeigt sich ein gänzlich anderes Muster: In Europa applizierten 22 % der Intensivstationen keinem Patient*in eine PN und 4 % allen Patient*innen, während außerhalb Europas 45 % der Stationen keinen Patient*in und 3 % alle Patient*innen mit PN versorgten.

Die Ernährungsform ist generell sehr stark von der Aufenthaltsdauer abhängig

**Figure 2:**
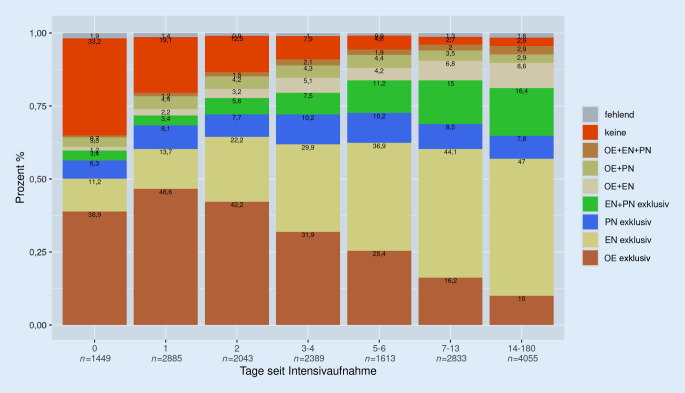

Die Ernährungsform ist generell sehr stark von der Aufenthaltsdauer (Abb. 2) abhängig. Dieses Abhängigkeitsmuster ergibt sich aus 2 einander ergänzenden Prozessen. Einerseits werden Patient*innen mit gewissen Ernährungsformen schneller oder langsamer entlassen und andererseits werden die Ernährungsformen im Lauf des Intensivaufenthalts angepasst. Besonders auffällig ist, dass insbesondere der Anteil an Patient*innen mit EN, aber auch mit PN mit zunehmender Aufenthaltsdauer zunimmt, während der Anteil mit OE stark abnimmt.

### Ernährungsform zum Entlasszeitpunkt

**Figure 3:**
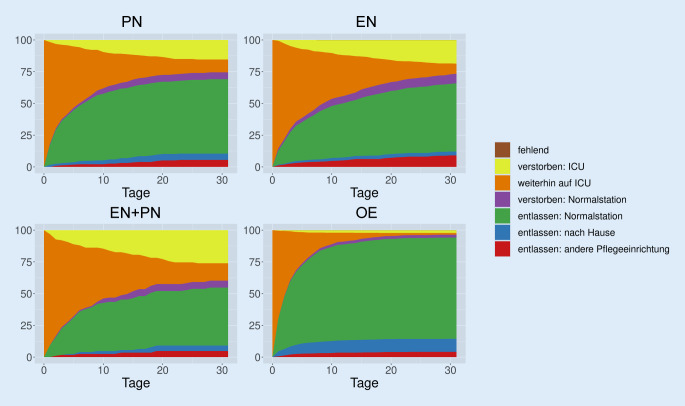

Das Entlassungsprofil wurde mathematisch aus den Daten rekonstruiert, um die tatsächlichen kumulativen Inzidenzen abzuschätzen und darstellen zu können. Eine OE ist mit der frühesten Entlassung aus der Intensivstation verbunden (Abb. 3: rot + blau + grün + violett) gefolgt von einer PN, während eine EN und insbesondere die Kombination aus EN und PN mit der spätesten Entlassung assoziiert sind. Patient*innen mit OE werden in 50 % nach 2 Tagen und in 80 % der Fälle nach einer Woche entlassen, Patient*innen mit PN in 30 % nach 2 Tagen und in etwas über 50 % nach einer Woche sowie Patient*innen mit EN in 20 % nach 2 Tagen und in etwa 45 % der Fälle nach einer Woche.

Der Anteil der Patient*innen mit sehr langer Intensivaufenthaltsdauer, die nach 30 Tagen noch auf der Intensivstation sind, ist am größten, wenn die Ernährung eine kombinierte Gabe aus EN und PN notwendig machte, deutlich geringer bei PN gefolgt von EN und OE (Abb. 3 orange). Dies spricht dafür, dass die Kombination aus EN und PN vor allem bei den schwerkranken Langliegern zum Einsatz kommt. Die Mortalität zeigt ein ähnliches Muster nach 30 Tagen wie auch am Ende der Beobachtungszeit nach 60 Tagen. In allen Weltregionen ist etwa ein Drittel der Patient*innen, die am nutritionDay auf der Intensivstation waren und eine EN erhielten, verstorben, minimal weniger Intensivpatient*innen verstarben mit Erhalt einer PN, aber viel seltener starben Intensivpatient*innen mit OE (Anteil von 10 %). Etwa ein Viertel der Patient*innen, die keine Ernährung am nutritionDay erhielten, waren verstorben.

### Praxis der medizinischen Ernährungstherapie

Eine Ernährungstherapie sowohl mit EN als auch PN beginnt weltweit und in Europa sehr ähnlich bei der Hälfte der Patient*innen am Tag 1 auf der Intensivstation, allerdings bei 25 % der Patient*innen erst nach Tag 4. Die DACH-Region unterscheidet sich geringfügig mit einem medianen EN-Beginn am Tag 2 bei gleicher Streuung und seltenerem spätem PN-Beginn am Tag 2,5 gegenüber den weltweiten und gesamteuropäischen Daten. Der Beginn der Ernährungstherapie wurde bei 7210 von 9099 Patient*innen mit EN und 3062 von 4505 mit PN dokumentiert.

Die Menge der verabreichten Ernährung (Tab. 2) war am nutritionDay mit etwa 1500 kcal im Median (normalisiert auf das Körpergewicht: 19 kcal/kgKG und Tag, IQR 12–26) unabhängig von der Weltregion und der Ernährungsform sehr ähnlich. Allerdings ist die Streuung mit 25 % der Patient*innen, die ein Viertel unter dem Median erhielten, und einem weiteren Viertel, das ein Drittel über Median erhielt, sehr groß. Der Unterschied zwischen geplanter und verabreichter Ernährung ist klein (Median 0, IQR −265 bis +100 kcal). Die Menge von geplanter bzw. verabreichter EN war bei 90 % bzw. 85 % der Patient*innen dokumentiert und bei 78 % bzw. 75 % der Patient*innen bei PN.

**Table 2:** 

	Weltweit		Europa		DACH	
N	18.918		11.179		1773	
	Median	IQR	Median	IQR	Median	IQR
N Tag	7210	–	4478	–	649	–
Tag EN-Beginn	1	0–4	1	0–4	2	0–4
*EN*						
Geplant (kcal/24 h)	1550	1200–1950	1584	1200–2000	1600	1200–2000
Verabreicht (kcal/24 h)	1474	960–1843	1500	1000–1900	1500	1000–1926
N Tag	3062	–	2432	–	492	–
Tag PN-Beginn	1	0–4	1	0–4	1	0–2,5
*PN*						
Geplant (kcal/24 h)	1560	1100–2000	1609	1138–2066	1560	1100–1978
Verabreicht (kcal/24 h)	1500	1000–1947	1500	1000–2000	1480	999–1891
PN	4505 (24 %)	–	3346 (30 %)	–	588 (33 %)	–
EN	9099 (48 %)	–	5511 (49 %)	–	760 (43 %)	–
OE	7363 (39 %)	–	4376 (39 %)	–	799 (45 %)	–
Keine Ernährung	1956 (10 %)	–	1040 (9 %)	–	197 (11 %)	–
GRV > 200 ml	806 (4,3 %)	–	619 (5,5 %)	–	108 (6,1 %)	–

*EN* enterale Ernährung; DACH Deutschland, Österreich, Schweiz;* GRV* gastrale Residualvolumen,* IQR* Interquartilsabstand,* OE* orale Ernährung;* PN* parenterale Ernährung

**Figure 4:**
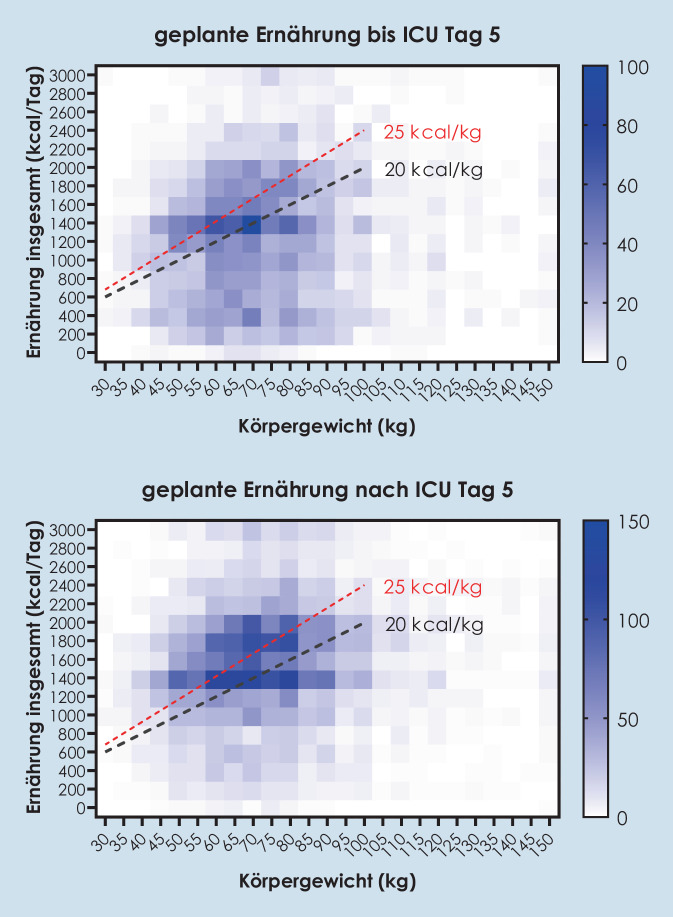

Die Beziehung zwischen dem Körpergewicht, dem zumeist empfohlenen Maß zur Schätzung des notwendigen Energiebedarfs und der tatsächlich geplanten Menge an Ernährung zeigt in keiner Weise die erwartete Beziehung, die einen Anstieg mit dem Körpergewicht und eine generelle Gruppierung um 20–25 kcal/kgKG und Tag erwarten lassen würde (Abb. 4). Die empfohlene Anpassung der Ernährungsmenge bei Adipositas würde einen flacheren Anstieg bei höherem Körpergewicht erwarten lassen. Der Unterschied zwischen den unterschiedlichen BMI-Gruppen ist relativ gering (Tab. 3). Frauen erhalten etwas weniger Ernährung im Vergleich mit Männern (*p* < 0,001).

**Table 3:** 

	Gesamte Ernährung		Proteinzufuhr	
	kcal/Tag (Median, IQR)	kcal/kgKG und Tag (Median; IQR)	g/Tag (Median; IQR)	g/kgKG und Tag (Median; IQR)
*BMI*				
<18,5	1230, 800–1600	28; 17–36	53; 35–72	1,14; 0,75–1,53
18,5–25	1440, 900–1800	22; 14–28	58; 36–78	0,91; 0,57–1,24
25–30	1500, 947–1890	19; 12–24	60; 38–80	0,78; 0,49–1,05
>30	1475, 925–1896	15; 9–19	60; 38–81	0,63; 0,37–0,85
*Geschlecht*				
Frauen	1350, 825–1725	19; 12–26	56; 35–76	0,82; 0,49–1,14
Männer	1500, 960–1896	19; 12–25	60; 38–82	0,80; 0,48–1,11

*IQR* Interquartilsabstand

Die Ernährungsmenge war unabhängig von der Weltregion und der Ernährungsform sehr ähnlich

Die auf das Körpergewicht bezogene Ernährungsmenge nimmt mit zunehmendem BMI ab (Tab. 3). In allen BMI-Gruppen ist die zugeführte Ernährungsmenge im obersten Quartil mehr als doppelt so hoch wie im untersten Quartil. Die Streuung ist etwas größer bei Patient*innen, die am nutritionDay in der frühen Phase auf der Intensivstation waren (≤ Tag 5). Dabei scheint es 2 Gruppen zu geben: eine mit niedriger Ernährungsmenge bei etwa 400–500 kcal und eine mit höhere Menge bei 1400–1500 kcal.

Die tägliche Proteinmenge betrug im Median 60 g (IQR 38–80) entsprechend einer Menge von 0,8 g/kgKG und Tag (IQR 0,5–1,1). Mit zunehmendem BMI bleibt die Menge sehr ähnlich (Tab. 3) Aufgrund der hohen Korrelation zwischen Ernährungsmenge und Protein findet man ebenfalls ein ähnliches Profil in Bezug auf den BMI.

## Resümee

Die Daten aus der Querschnittskohorte von Intensivpatient*innen des nutritionDay stellen die größte prospektive Stichprobe von Ernährungsdaten aus 63 Ländern dar und spiegeln ein Bild der täglichen Praxis wider. Dabei zeigt sich, dass bei der Hälfte der Patient*innen am Tag 1 mit einer Ernährungstherapie begonnen wird und dass ab Tag 3 über 90 % der Patient*innen ernährt sind. Die Menge der verabreichten Ernährung erscheint im Median nahezu den Empfehlungen mit 19 kcal/kgKG und Tag und der generell von der WHO empfohlenen Proteinmenge mit 0,8 g/kgKG und Tag zu entsprechen, obwohl die Empfehlungen für Protein bei Intensivpatient*innen deutlich höher liegen. Ähnliche Beobachtungen wurden rezent in Europa auf 77 unterschiedlichen Intensivstationen gemacht [20].

Bemerkenswert ist die breite Streuung der Menge der verabreichten Ernährung, die kaum Anpassung an das Körpergewicht erkennen lässt. Diese Streuung bleibt bestehen, wenn die zugeführten Mengen durch das Körpergewicht dividiert werden, weil dadurch verschleiert wird, dass die gering ernährten Patient*innen eher adipös und die übermäßig ernährten eher mager sind.

Die beobachtete Änderung der Ernährungsformen mit zunehmender Aufenthaltsdauer wurde in sehr ähnlicher Weise bisher nur für Europa in einer rezenten großen Beobachtungsstudie von 1172 Patient*innen beschrieben [20]. Der Vergleich der verschiedenen Weltregionen zeigt klare Unterschiede für die Anwendung von PN, die außerhalb von Europa deutlich seltener verwendet wird.

Limitationen des nutritionDay-Projekts sind die freiwillige Teilnahme, die nicht alle Länder entsprechend ihrer Größe repräsentiert, und ein Selektionsbias von Intensivstationen mit einem vermehrten Interesse an Ernährungstherapie. Eine weitere Limitation ergibt sich aus der Querschnittsuntersuchung, die zu einer größeren Wahrscheinlichkeit der Rekrutierung von länger liegenden Patient*innen und damit schwerer kranken Patient*innen führt. Für die aktuelle Untersuchung zur Beschreibung der Ernährungstherapie wurde dieser Tatsache durch entsprechende Berücksichtigung der Aufenthaltsdauer, wenn angebracht, oder durch entsprechende Gewichtung oder mathematische Rekonstruktion des inzidenten Verlaufs aus den prävalenten Beobachtungen Rechnung getragen [28, 29].

## Fazit für die Praxis

- Die im Rahmen der nutritionDay-Kohorte in den Jahren 2007–2021 auf den Intensivstationen erhobenen Daten zeigen, dass die Ernährungstherapie den meisten Patient*innen zugutekommt.
- Bei vielen Patient*innen erfolgt in den ersten Tagen nach Aufnahme auf die Intensivstation kein progressiver Ernährungsaufbau.
- Die Ernährung wird vielfach nicht sehr personalisiert, z. B. nicht an das Körpergewicht abgepasst, verabreicht.
- Die applizierte Ernährungsmenge ist in allen Weltregionen sehr ähnlich und zeigt eine breite Streuung.
- Mit zunehmender Aufenthaltsdauer auf der Intensivstation nimmt der Anteil von Patient*innen mit oraler Ernährung ab und mit parenteraler sowie enteraler Ernährung zu.

## References

[1] Compher C, Bingham AL, McCall M, Patel J, Rice TW, Braunschweig C (2022). Guidelines for the provision of nutrition support therapy in the adult critically ill patient: The American Society for Parenteral and Enteral Nutrition. Jpen J Parenter Enteral Nutr.

[2] Weimann A, Braga M, Carli F, Higashiguchi T, Hubner M, Klek S (2021). ESPEN practical guideline: clinical nutrition in surgery. Clin Nutr.

[3] Singer P, Blaser AR, Berger MM, Alhazzani W, Calder PC, Casaer MP (2019). ESPEN guideline on clinical nutrition in the intensive care unit. Clin Nutr.

[4] McClave SA, Taylor BE, Martindale RG, Warren MM, Johnson DR, Braunschweig C (2016). Guidelines for the provision and assessment of nutrition support therapy in the adult critically ill patient: society of critical care medicine (SCCM) and American society for Parenteral and Enteral nutrition (A.S.P.E.N.). JPEN J Parenter Enteral Nutr.

[5] Reintam Blaser A, Starkopf J, Alhazzani W, Berger MM, Casaer MP, Deane AM (2017). Early enteral nutrition in critically ill patients: ESICM clinical practice guidelines. Intensive Care Med.

[6] Kumpf VJ, de Aguilar-Nascimento JE, Diaz-Pizarro JI, Hall AM, McKeever L, Steiger E (2017). ASPEN-FELANPE clinical guidelines. JPEN J Parenter Enteral Nutr.

[7] Singer P, Berger MM, Van den Berghe G, Biolo G, Calder P, Forbes A (2009). ESPEN guidelines on parenteral nutrition: intensive care. Clin Nutr.

[8] Weimann A, Braga M, Harsanyi L, Laviano A, Ljungqvist O, Soeters P (2006). ESPEN guidelines on enteral nutrition: surgery including organ transplantation. Clin Nutr.

[9] Kreymann KG, Berger MM, Deutz NE, Hiesmayr M, Jolliet P, Kazandjiev G (2006). ESPEN guidelines on enteral nutrition: intensive care. Clin Nutr.

[10] Casaer MP, Mesotten D, Hermans G, Wouters PJ, Schetz M, Meyfroidt G (2011). Early versus late parenteral nutrition in critically ill adults. N Engl J Med.

[11] Heyland DK, Dhaliwal R, Day AG, Muscedere J, Drover J, Suchner U (2006). REducing Deaths due to OXidative Stress (The REDOXS Study): Rationale and study design for a randomized trial of glutamine and antioxidant supplementation in critically-ill patients. Proc Nutr Soc.

[12] Singer P, Anbar R, Cohen J, Shapiro H, Shalita-Chesner M, Lev S (2011). The tight calorie control study (TICACOS): a prospective, randomized, controlled pilot study of nutritional support in critically ill patients. Intensive Care Med.

[13] Heidegger CP, Berger MM, Graf S, Zingg W, Darmon P, Costanza MC (2013). Optimisation of energy provision with supplemental parenteral nutrition in critically ill patients: a randomised controlled clinical trial. Lancet.

[14] Reignier J, Boisrame-Helms J, Brisard L, Lascarrou JB, Ait Hssain A, Anguel N (2018). Enteral versus parenteral early nutrition in ventilated adults with shock: a randomised, controlled, multicentre, open-label, parallel-group study (NUTRIREA-2). Lancet.

[15] Harvey SE, Parrott F, Harrison DA, Bear DE, Segaran E, Beale R (2014). Trial of the route of early nutritional support in critically ill adults. N Engl J Med.

[16] Rice TW, Wheeler AP, Thompson BT, Steingrub J, National Heart L, Blood Institute Acute Respiratory Distress Syndrome Clinical Trials N (2012). Initial trophic vs full enteral feeding in patients with acute lung injury: the EDEN randomized trial. JAMA.

[17] Doig GS, Simpson F, Sweetman EA, Finfer SR, Cooper DJ, Heighes PT (2013). Early parenteral nutrition in critically ill patients with short-term relative contraindications to early enteral nutrition: a randomized controlled trial. JAMA.

[18] Heyland D, Muscedere J, Wischmeyer PE, Cook D, Jones G, Albert M (2013). A randomized trial of glutamine and antioxidants in critically ill patients. N Engl J Med.

[19] Arabi YM, Aldawood AS, Haddad SH, Al-Dorzi HM, Tamim HM, Jones G (2015). Permissive underfeeding or standard enteral feeding in critically ill adults. N Engl J Med.

[20] Matejovic M, Huet O, Dams K, Elke G, Vaquerizo AC, Csomos A (2022). Medical nutrition therapy and clinical outcomes in critically ill adults: a European multinational, prospective observational cohort study (EuroPN). Crit Care.

[21] Elke G, Hartl WH, Kreymann KG, Adolph M, Felbinger TW, Graf T (2019). DGEM Guideline “Clinical Nutrition in Critical Care Medicine”—short version. Anasthesiol Intensivmed Notfallmed Schmerzther.

[22] https://www.nutritionday.org/en/30-languages/languages.html

[23] Vincent JL, Moreno R, Takala J, Willatts S, De Mendonca A, Bruining H (1996). The SOFA (Sepsis-related Organ Failure Assessment) score to describe organ dysfunction/failure. On behalf of the Working Group on Sepsis-Related Problems of the European Society of Intensive Care Medicine. Intensive Care Med.

[24] Le Gall JR, Lemeshow S, Saulnier F (1993). A new Simplified Acute Physiology Score (SAPS II) based on a European/North American multicenter study. JAMA.

[25] Reis Miranda D, Moreno R, Iapichino G (1997). Nine equivalents of nursing manpower use score (NEMS). Intensive Care Med.

[26] https://www.nutritionday.org/cms/upload/pdf/6_about_nutritionDay/6.1.example_reports/ICU_example_report.pdf

[27] Sulz I, Bauer P, Hiesmayr M (2020). Estimating outcome without cross-sectional bias in ICU nutritionday. Clin Nutr ESPEN.

[28] Frantal S, Pernicka E, Hiesmayr M, Schindler K, Bauer P (2016). Length bias correction in one-day cross-sectional assessments—the nutritionDay study. Clin Nutr.

[29] Veraar C, Geilen J, Fischer A, Sulz I, Tarantino S, Mouhieddine M (2021). Timing of parenteral nutrition in ICU patients: a transatlantic controversy. Clin Nutr ESPEN.

